# A Review of Controversial Issues in the Management of Head and Neck Cancer: A Swiss Multidisciplinary and Multi-Institutional Patterns of Care Study—Part 4 (Biomarkers)

**DOI:** 10.3389/fonc.2019.01128

**Published:** 2019-10-24

**Authors:** Martina A. Broglie, Pavel Dulguerov, Guido Henke, Marco Siano, Paul Martin Putora, Christian Simon, Daniel Zwahlen, Gerhard F. Huber, Giorgio Ballerini, Lorenza Beffa, Roland Giger, Sacha Rothschild, Sandro V. Negri, Olgun Elicin

**Affiliations:** ^1^Department of Otorhinolaryngology, Head and Neck Surgery, Cantonal Hospital St. Gallen, St. Gallen, Switzerland; ^2^Department of Otorhinolaryngology, Head and Neck Surgery, University Hospital Zurich, Zurich, Switzerland; ^3^Department of Otorhinolaryngology, Head and Neck Surgery, Geneva University Hospital, Geneva, Switzerland; ^4^Department of Radiation Oncology, Cantonal Hospital St. Gallen, St. Gallen, Switzerland; ^5^Department of Medical Oncology, Cantonal Hospital St. Gallen, St. Gallen, Switzerland; ^6^Department of Medical Oncology, Hôpital Riviera-Chablais, Vevey, Switzerland; ^7^Department of Radiation Oncology, Inselspital, Bern University Hospital, University of Bern, Bern, Switzerland; ^8^Department of Otorhinolaryngology, Head and Neck Surgery, University Hospital of Lausanne, Lausanne, Switzerland; ^9^Department of Radiation Oncology, Cantonal Hospital Graubünden, Chur, Switzerland; ^10^Department of Radiation Oncology, Cantonal Hospital of Winterthur, Winterthur, Switzerland; ^11^Department of Radiation Oncology, Clinica Luganese SA, Lugano, Switzerland; ^12^Department of Radiation Oncology, Cantonal Hospital Lucerne, Lucerne, Switzerland; ^13^Department of Otorhinolaryngology, Head and Neck Surgery, Inselspital, Bern University Hospital, Bern, Switzerland; ^14^Department of Medical Oncology, University Hospital of Basel, Basel, Switzerland; ^15^Department of Otorhinolaryngology, Lindenhofspital, Bern, Switzerland

**Keywords:** consensus, head and neck cancer, patterns of care, practice patterns, survey

## Abstract

**Background:** The Head and Neck Cancer Working Group of Swiss Group for Clinical Cancer Research (SAKK) has investigated the level of consensus (LOC) and discrepancy in everyday practice of diagnosis and treatment in head and neck cancer.

**Materials and Methods:** An online survey was iteratively generated with 10 Swiss university and teaching hospitals. LOC below 50% was defined as no agreement, while higher LOC were arbitrarily categorized as low (51–74%), moderate (75–84%), and high (≥85%).

**Results:** Any LOC was achieved in 62% of topics (*n* = 60). High, moderate, and low LOC were found in 18, 20, and 23%, respectively. Regarding Head and Neck Surgery, Radiation Oncology, Medical Oncology, and biomarkers, LOC was achieved in 50, 57, 83, and 43%, respectively.

**Conclusions:** Consensus on clinical topics is rather low for surgeons and radiation oncologists. The questions discussed might highlight discrepancies, stimulate standardization of practice, and prioritize topics for future clinical research.

## Introduction

This is the fourth part of the article “A Review of Controversial Issues in the Management of Head and Neck Cancer: A Swiss Multidisciplinary and Multi-Institutional Patterns of Care Study,” providing the results for the items concerning biomarkers, each followed by a short discussion if deemed relevant. The details of the methodology is presented in the first part of this series.

## Results and Discussion of Biomarkers with Current Potential Use

➢ *Imaging findings indicating hypoxia and/or central necrosis are not standard factors influencing the treatment decision: no consensus*.

In 5/10 centers, imaging findings indicating hypoxia or central necrosis affects the decision for the primary treatment modality.

According to the literature (1), tumor hypoxia can be associated with aggressive tumor phenotype affecting the natural course of disease in these patients (2) due to assumed radiotherapy resistance. Based on laboratory experience, a up to three times higher photon radiation dose is needed to cause the same cytotoxic effect in hypoxic cells compared to normal tumor cells (3). Whether such dose escalation could be performed, while keeping low toxicity rates in normal tissue is questionable (4). The advantage of dose escalation to hypoxic sub-volumes with conventional photon radiation has been analyzed in clinical practice to overcome this bad prognostic factor (5–8). However, the clinical identification, measurement, and localization of hypoxia in tumors remain debatable. The studies range from non-invasive clinical assumptions to direct measurements with oxygen electrodes, and indirect methods such as serum biomarkers or immunohistochemistry (IHC) of hypoxia-related markers. There has been found a significant heterogeneity in regional oxygenation as well as in biological response to hypoxia confounding these tissue-sampling methods. In current clinical practice, boost dose is guided by CT scans and is based primarily on size criteria (1). However, the correlation between tumor hypoxia and common clinical parameters such as size, morphology, and histology is scarce (9). PET scans could deliver more functional information based on tumor metabolism (10).

In tumors with presence of diffuse hypoxia a systemic approach using a hypoxic cell cytotoxin or anti-growth factor drugs might be beneficial to overcome the limitations of hypoxia (11). Alternatively, in a more focal hypoxia a local/regional approach, such as IMRT-based radiation dose escalation to the hypoxic sub-volume might be more successful (12, 13). In different studies, the complementary role of radiation and systemic hypoxia-specific pro-drugs to overcome the hypoxia-induced resistance has been established (14, 15). Furthermore, there is a higher risk for persistence of hypoxic tumors after primary CRT and the timing of salvage surgery such as planned neck dissection should be adapted.

Anyhow, regarding the limited evidence for the role of imaging findings indicating hypoxia, it is quite remarkable that half of the centers in Switzerland integrate them in treatment decisions.

➢ *De-differentiation grade is not a standard factor influencing the treatment decision: no consensus*.

De-differentiation grade of tumors also influences treatment decision in 5/10 centers. This question did not differ between squamous cell cancer and other malignancies of the head and neck.

In salivary gland carcinoma, the histologic grade is a significant predictor of treatment response and an established factor for therapeutic decisions, but due to the rarity and wide variety of different tumor types the definition of predictive grading schemes is challenging (16).

In HNSCC, histologic grade is not part of the current staging criteria, probably because its prognostic impact remains controversial. Weijers et al. (17) found no significant correlation between grade and prognosis in early stage oral cancer. In contrast, other studies (18–20) found a significant impact of tumor differentiation and staging on recurrence and overall survival. Furthermore, a recent study (21) in early stage oral cancer has demonstrated a strong association between histologic grade and survival. High histologic grade was associated with poorer survival and carried an independent prognostic value in addition to tumor size, node status, and presence of distant metastasis (TNM) stage (21). Even though grade is not part of the UICC staging system, some centers do consider high grade as an indication for adjuvant treatment (22, 23).

➢ *Determining the HPV status is a standard practice in oropharyngeal squamous cell carcinoma (OPSCC): high LOC (100%)*.
➢ *There is no standard method established for the definition of HPV status: no consensus*.

All (10/10) centers regularly determine the HPV status in OPSCC. The definition of an HPV attributable tumor is IHC overexpression of p16 as a single marker (5 centers), HPV high-risk type DNA positivity by polymerase chain reaction (PCR) (2 centers); HPV high risk type DNA positivity by ISH (1 center) and p16, followed by PCR if needed according to College of American Pathologists guidelines (1 center).

The survey was performed prior to the release of the 8th edition of the UICC TNM classification system, implementing p16 IHC as a crucial biomarker for staging of OPSCC. Nevertheless, all centers had already started to routinely determine the HPV-status in OPSCC. Interestingly, the definition of a positive HPV-status widely differs between the centers, reflecting the lack of a worldwide-accepted consensus for the accurate definition of an HPV-driven cancer. In the new UICC staging system (8th edition), p16 is accepted for practical and cost-related reasons considering the guideline to be international (24), but the definition of a high-risk HPV-attributed cancer is still a matter of debate (25).

Currently, detection of p16^INK4A^ (inhibitor of cyclin-dependent kinase 4) overexpression in tumor tissue by IHC is used as a surrogate marker for HPV-driven HNSCC (26). However, p16^INK4A^ IHC as a single diagnostic marker has shown insufficient sensitivity (27–30) and specificity (27, 29–31).

Due to its high sensitivity, high-risk HPV-DNA detection by quantitative PCR has been commonly employed to detect HPV-driven tumors, but was found to lack sufficient specificity, which could lead to false positive results (32). Indeed, HPV-DNA detection in tumor specimens is not proving a causal viral association of carcinogenesis but could also be the result of a past non-transforming HPV-infection or contamination (33). Detection of the transcripts of viral oncogenes E6 and E7 in tumor through mRNA techniques is widely accepted as gold standard for determining the oncogenic role of HPV in tumor. However, extraction techniques and analyses of RNA from the routinely available formalin-fixed paraffin-embedded tissue specimens remain challenging and costly, limiting their widespread use (27). In this context, Smeets et al. (31) validated an algorithm based on the combination of p16^INK4A^ IHC followed by HPV-DNA analysis to detect an oncogenically active HPV infection in formalin-fixed paraffin-embedded tissue specimens: the accuracy, sensitivity, and specificity were 98, 96, and 98%, respectively when compared to RNA detection (34), that is why it would probably be the most suitable definition for tumoral HPV-association in clinical routine.

➢ *Most centers determine the HPV status in a carcinoma of unknown primary (CUP): low LOC (60%)*.

In 6/10 centers, HPV status is routinely determined in lymph node metastases without evidence of a primary tumor.

Cervical lymph node metastases from clinically undetectable primary squamous cell carcinoma present a diagnostic and therapeutic challenge. There is no clear consensus for the optimal treatment in CUP. Recommendations range from surgery of the neck alone to primary radiotherapy of the mucosa at risk and both neck sides (35–39). In the era of treatment de-intensification, the potential benefit of radiotherapy of putative primary tumor sites has to be weighed against its detrimental effect on quality of life and additional toxicity. The role of high-risk HPV infection in the development of HNSCC has gained evidence (40, 41), in particular for CUP. Several studies showed a high correlation between HPV-positive lymph node metastases and the detection of the primary tumor in the oropharynx. Many HPV-associated tumors present with prominent nodal disease and small, difficult or even undetectable (clinically and radiologically) primaries hidden in palatine and lingual tonsillar crypts (42). Therefore, a rising incidence of HPV-positive lymph node metastases manifesting as CUP has been reported (43–46). Unfortunately the sensitivity of ^18^FDG-PET/CT is adversely affected by false positives from hypermetabolic oropharyngeal lymphoid tissue (42). Even in patients with CUP HPV-positivity in lymph node metastases is a positive prognostic factor and influencing treatment decisions (47). This was accounted for in the updated 8th edition of the UICC classification by integrating HPV-positivity of the primary tumor or the lymph node metastases in CUP staging. Since the survey was performed prior to the release of the 8th edition of the UICC TNM classification system it is interesting to see, that at that time the importance of HPV infection in CUP was not evident in 40% of the centers in Switzerland.

After an intensive literature search in Pubmed and Medline we have only found one comparable survey about patterns of care for CUP. It has been performed recently in Germany, only included radiation oncologists and has revealed that 82% of the departments routinely determined HPV status in CUP (48). This rate is significantly higher than in Switzerland. According to the authors it is explained by the requirements to stage a CUP according to the 8th TNM-classification edition and known as an increasingly important prognostic factor.

➢ *Determination of the HPV status in non-oropharyngeal HNSCC is not accepted as a standard practice: no consensus*.

HPV status is also determined in non-oropharyngeal primaries in 4/10 centers.

This question is related to whether the presence of HPV in non-oropharyngeal HNSCC represent viral-mediated carcinogenesis, or merely a “bystander” infection and whether HPV-positivity in such cases influences the treatment strategy and clinical outcome (49). Large data on HPV DNA detection by PCR and p16 expression in HNSCC biopsies suggests that the probability of a cancer of the oral cavity, larynx, and hypopharynx being attributable to HPV is at least 5-fold lower than that for OPSCC (49, 50). High-risk HPV DNA was also detected in a significant proportion of sinonasal, nasopharyngeal, and salivary gland cancers, but the clinical significance of these findings in these malignancies has not been clearly defined. Limited data on HPV E6/E7 mRNA suggests that HPV-attributable HNSCC is rare in the oral cavity (3%), larynx (7%) and lacking in the hypopharynx (0%). Concerning the prognostic impact of HPV-positivity in non-oropharyngeal subsites, no data currently supports that HPV is significantly associated with improved outcome in oral or laryngeal cancer (49), while data are lacking for other subsites (49). In the absence of appropriately powered, well-designed studies, HPV-detection in non-oropharyngeal sites does not seem to impact staging or treatment.

➢ *Most centers do not base their treatment decision on HPV status: moderate LOC (80%)*.

HPV status does not influence the treatment decision in 8/10 centers. Two centers stated that they may consider changing the treatment intensity.

Although HPV-positivity in OPSCC is an established positive prognostic marker, treatment decisions should so far not be influenced by it. There is a lot of ongoing discussion about treatment de-escalation in this low-risk tumor but centers should wait for the shortcoming results of prospective clinical trials to decide on less intensive treatment regimen.

## Summary

In summary, there is no consensus regarding the applicability of imaging findings indicating hypoxia as well as histological differentiation grade. In all centers, the determination of the HPV-status is a standard practice in oropharyngeal squamous cell carcinoma rather than in cancer of unknown primary. Since the survey was performed prior to the release of the 8th edition of the UICC TNM classification system it is interesting to see, that at that time the importance of HPV infection especially in CUP was not evident in almost half of the centers in Switzerland.

Furthermore, there is a lack of standard method established for the definition of HPV-status ranging from p16 IHC as a single marker, HPV-DNA by PCR or ISH as single markers or the combination of both reflecting the lack of a worldwide-accepted consensus for the accurate definition of an HPV-driven cancer. In the majority of centers, there is no therapeutic consequence of HPV-testing in both OPSCC and CUP due to lack of practice guidelines based on prospective clinical trials.

## Conclusion

The findings of our survey indicate a low LOC among head and neck oncologists working in academic and multidisciplinary setting in 10 Swiss institutions. The highest LOC was achieved among medical oncologists, whereas the lowest was observed among head and neck surgeons. On the other hand, this level of disagreement may also depend on the topics chosen for the survey, and not necessarily the heterogeneity within the disciplines. It is also interesting to witness a low LOC regarding topics, where a high level of evidence actually does exist, and vice versa. This article is expected to serve the head and neck oncologists to be aware of their discrepancies and to stimulate discussion toward standardization of practice and prioritize topics of future clinical research.

## Data Availability Statement

All datasets generated for this study are included in the manuscript/supplementary files.

## Author Contributions

GH, MB, OE, PD, and PP: conception and design. OE and PP: collection of data. Generation of the initial and final versions of the questions, drafting of the manuscript, and approval of the final version by all co-authors.

### Conflict of Interest

The authors declare that the research was conducted in the absence of any commercial or financial relationships that could be construed as a potential conflict of interest.
